# Supportive Treatment of a Dog with Leishmaniosis and Severe Glomerulopathy with Immunoadsorption

**DOI:** 10.3390/pathogens13030193

**Published:** 2024-02-21

**Authors:** Florian Sänger, Michèle Bergmann, Katrin Hartmann, René Dörfelt

**Affiliations:** LMU Small Animal Clinic, Centre for Clinical Veterinary Medicine, Faculty of Veterinary Medicine, Ludwig-Maximilians-Universität München, 80539 Munich, Germany; n.bergmann@medizinische-kleintierklinik.de (M.B.); hartmann@lmu.de (K.H.); r.doerfelt@medizinische-kleintierklinik.de (R.D.)

**Keywords:** azotemia, proteinuria, immunoglobulin G, immunoglobulin M, immunocomplex glomerulonephritis, leishmania infantum infection

## Abstract

A three-year-old, intact female mix-breed dog, weighing 30 kg, was presented due to vomitus and diarrhea. At presentation, the patient had a slightly reduced general condition and moderately enlarged mandibular and popliteal lymph nodes. The initial blood work showed severe azotemia and hypoalbuminemia. In the urinalysis, marked proteinuria with a urine protein/creatinine ratio (UPC) of 4.69 was found. Further workup showed a high leishmania antibody titer. The dog was diagnosed with leishmaniosis and glomerulonephritis. Initial treatment consisted of intravenous fluid therapy, allopurinol, miltefosine, amlodipine, clopidogrel, and a diet with a low purine content. Creatinine temporarily decreased but increased again after three days. For further supportive treatment, intermittent hemodialysis in combination with hemoperfusion with the cytosorb^®^ adsorber was performed. A total blood volume of 17.7 L was processed within three hours. Thereafter, immunoadsorption (IA) was performed with the COM.TEC^®^ and ADAsorb^®^ platforms and a LIGASORB^®^ adsorber to eliminate circulating immunocomplexes. Treatment time for IA was two hours with a blood flow of 50 mL/min. A total plasma volume of 2.4 L was processed. Over the following days, creatinine declined, and the patient improved significantly. UPC decreased to 1.74 on day 17 after IA. The patient was discharged after two and a half weeks. Two years after the initial event, the patient is still in excellent condition, with creatinine, UPC, and albumin levels in the reference range. Therefore, IA might be an additional therapeutic option for dogs with leishmaniosis-induced glomerulonephritis and subsequent severe azotemia to improve immunocomplex-mediated glomerulonephritis.

## 1. Introduction

Canine leishmaniosis has become one of the most important emerging diseases in dogs over the last few years. It is a vector-borne disease caused by the protozoon *Leishmania infantum* that is transmitted by sandflies (*Phlebotomus)* [1]. Canine leishmaniosis is a chronic and multisystemic disease, and patients can show cutaneous (e.g., non-itchy diffused alopecia, hyperkeratosis, ulcers, secondary infections) and/or systemic signs (e.g., weight loss, hyporexia, generalized muscle atrophy) and visceral signs (e.g., diarrhea due to granulomatous or pyogranulomatous intestinal inflammation, signs of uveitis and polyarthritis) [1,2]. The majority of clinical signs are caused by the deposition of immunocomplexes (immunoglobulin G (IgG) and immunoglobulin M (IgM)) in the different organs, especially in the kidneys, blood vessels, joints, and skin [2]. Standard therapy includes leishmanicidal therapy with meglumine antimonate or miltefosine in combination with leishmanistatic therapy with allopurinol and a purine-reduced diet [1,2]. Further supportive treatment depends on the patient’s individual alterations. Usually, therapy leads to clinical improvement of the disease, but complete elimination of the pathogen (usually) cannot be achieved [3]. Therefore, many patients remain infected, and relapses of disease can occur [2]. The progression of glomerulonephritis and proteinuria leads to hypertension and tubular kidney disease, which can ultimately develop into chronic tubulointerstitial kidney disease or nephrotic syndrome, the most common cause of death in patients with leishmaniosis [1,4].

Immunoadsorption (IA) is an extracorporeal blood purification technique that is used to eliminate antibodies and circulating immunocomplexes from the blood. In human medicine, IA is a well-described treatment option for many immunological diseases, such as autoimmune hemolytic anemia, immune-mediated thrombocytopenia, systemic lupus erythematosus, immunocomplex glomerulonephritis, or myasthenia gravis [5]. In veterinary medicine, no studies currently prove the efficacy of IA in dogs. To the authors’ knowledge, this is the first report that describes the use of IA in a canine patient with leishmaniosis.

## 2. Case Presentation

A three-year-old intact female mixed-breed dog, weighing 30 kg, was presented due to vomitus and diarrhea for two days. Initially, the dog was imported from Mallorca, Spain, as a young dog. In addition, the dog had been to France the year before the presentation. Routine blood work at the referring veterinarian showed significant azotemia (creatinine 602 µmol/L; reference (ref.) range: 44–125 µmol/L). The dog was referred for further diagnostics and treatment of a suspected acute kidney injury. During the initial physical examination, the patient had a slightly reduced general condition, a heart rate of 100/min, pink mucous membranes with a capillary refill time of less than two seconds, a rectal temperature of 38.7 °C, and moderately enlarged mandibular and popliteal lymph nodes. The initial blood work (blood gas and hematology (CBC)) was unremarkable: severe azotemia with increased creatinine (611 µmol/L; ref. range: 44–125 µmol/L) and urea (32.1 mmol/L; ref. range: 3.5–10.8 mmol/L) and hypoalbuminemia (24.9 g/L; ref. range: 31.3–43.0 g/L). In the urinalysis, marked proteinuria with a urine protein/creatinine ratio (UPC) of 4.69 was present. Urine sediment was unremarkable. For further workup, an abdominal ultrasound, a bacterial culture of the urine, leptospirosis PCR in urine and blood, a microagglutination test for *Leptospira* ssp. antibodies, and a profile for vector-borne diseases (antibodies for *Ehrlichia canis*, *Babesia canis*, and *Leishmania* spp., and PCR for *Anaplasma* spp., *Hepatozoon canis*, microfilaria, and *Mycoplasma* spp.) in a commercial laboratory were initiated. On abdominal ultrasound, enlarged kidneys with a medullary rim sign without pyelectasia were observed. Bacterial culture and PCR for *Leptospira* spp. were negative. Tests for vector-borne diseases were all negative except for a positive *leishmania* antibody test (82.5 Units; ref. range: <7 Units). Therefore, the diagnosis of leishmaniosis was made.

**Figure 1:**
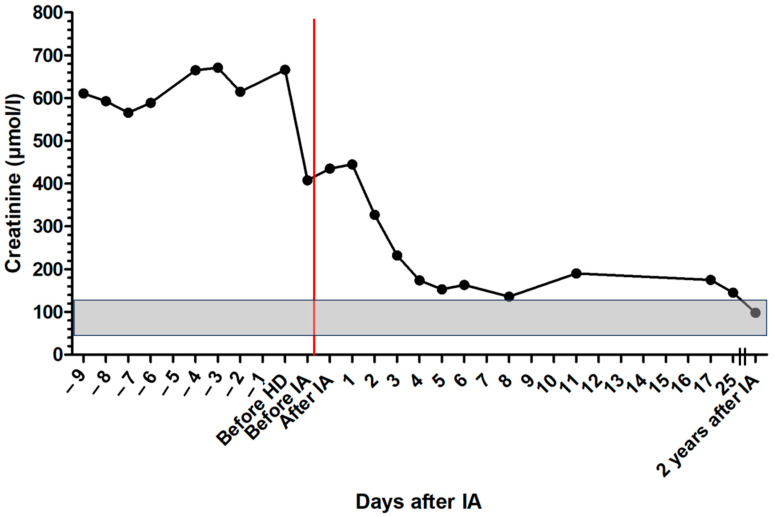
Course of creatinine concentration in the patient with leishmaniosis during treatment; the gray area marks the reference range, the red line marks the time point of immunoadsorption; HD = hemodialysis; IA = immunoadsorption.

**Figure 2:**
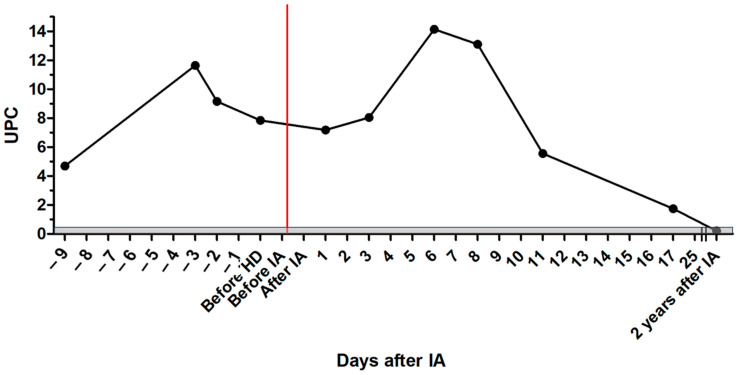
Course of UPC values in the patient with leishmaniosis during treatment; the gray area marks the reference range; the red line marks the time point of immunoadsorption; HD = hemodialysis; IA = immunoadsorption; UPC = urine protein/creatinine ratio.

Initial treatment consisted of intravenous fluid therapy, maropitant (1 mg/kg IV q 24 h; Prevomax, Dechra Pharmaceuticals, Northwich, UK), esomeprazole (1 mg/kg IV q 12 h; Nexium, Grünenthal, Aachen, Germany), antibiotic therapy with amoxicillin clavulanic acid (20 mg/kg IV q 8 h; Amoxiclav Hexal 500/100 mg, Hexal, Holzkirchen, Germany) until tests for leptospirosis were negative, and sucralfate (30 mg/kg PO q 8 h; Sucrabest 1 g granulate, Combustin, Hailtingen, Germany). After the diagnosis of leishmaniosis was made, the patient was treated with allopurinol (10 mg/kg PO q 12 h; Allopurinol-ratiopharm 100 mg, Ratiopharm, Ulm, Germany) and miltefosine (2 mg/kg PO q 24 h; Milteforan 20 mg/mL, Virbac, Carros, Italy). In addition, the dog received amlodipine (0.2 mg/kg PO q 12 h; Amodip 1.25 mg, Ceva, Brussel, Belgium) and clopidogrel (3 mg/kg PO q 24 h; Clopidogrel 75 mg, AAA-Pharma, Böblingen, Germany), and a purine-reduced diet (calculated self-cooked ration) was started. Creatinine decreased over the next two days. After that, the patient clinically deteriorated, creatinine increased again, and UPC increased to 11.65 (Figure 1 and Figure 2).

The deterioration of creatinine and UPC was most likely caused by leishmaniosis-induced potential glomerulonephritis. Therefore, IA to reduce immunocomplexes in this patient was started. IA was combined with hemodialysis and Cytosorb^®^ hemoperfusion to reduce azotemia and cytokine load. For this treatment, a 14 French, 25 cm double-lumen central venous catheter (Arrow Germany GmbH, Erding, Germany) was inserted in the right jugular vein. Intermittent hemodialysis in combination with hemoperfusion with a hemoperfusion adsorber (CytoSorb^®^, Cytosorbens Europe GmbH, Berlin, Germany) was performed. For hemodialysis, the dialysis platform Fresenius 4008 (Fresenius Medical Care GmbH und Co KG, Bad Homburg, Germany) was used, and a total blood volume of 17.7 L was processed during 3 h. Anticoagulation was performed with heparin (Heparin-Natrium 5000 IU/mL, B. Braun Melsungen GmbH, Melsungen, Germany) as a bolus of 60 IU/kg intravenously, followed by a constant-rate infusion of 60–80 IU/kg/h.

**Figure 3:**
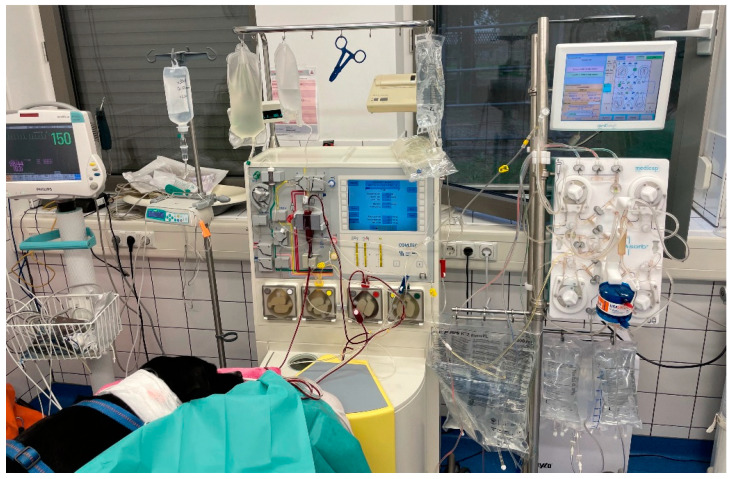
Setup of immunoadsorption for the dog with leishmaniosis.

Within 1 h after hemodialysis and hemoperfusion, IA was performed (Figure 3). Continuous plasma separation was performed via centrifugation (COM.TEC, Fresenius Kabi, Deutschland GmbH, Bad Homburg, Germany) using a commercial tubing system (P1R 9 400 411, Fresenius Kabi GmbH, Bad Homburg, Germany). Plasma was transferred to the connected immunoadsorption machine (ADAsorb, Medicap clinic GmbH, Ulrichstein, Germany) equipped with a tubing system (ADAsorb-LIGASORB Set, Medicap clinic GmbH, Ulrichstein, Germany) and a staphylococcus antitoxin A column (LIGASORB, Fresenius Medical Care Deutschland GmbH, Bad Homburg, Germany). Extracorporeal blood volume was 405 mL. Tubing was used and filled according to the manufacturer’s instructions with isotonic saline. Anticoagulation was performed with citrate (ACD-A, Fresenius Kabi, Bad Homburg, Germany) at a blood/citrate ratio of 1/14 in the beginning, with a stepwise reduction to 1/24. Additionally, heparin was given as a bolus of 30 IU/kg intravenously every hour. The dog received an infusion with calcium gluconate 10% (Calciumgluconat 10%, B. Braun Melsungen GmbH, Melsungen, Germany) via a peripheral venous catheter to avoid hypocalcemia, according to a protocol established for hemodialysis. Machine settings and blood flow of 50 mL/min were selected to process 2.4 L of plasma in four immunoadsorption cycles with 600 mL each (two-fold plasma volume). The dog was monitored during both procedures with electrocardiography, pulse oximetry, and non-invasive blood pressure with a multi-parameter monitor (Nihon Kohden, Tokyo, Japan). During hemodialysis, the dog showed phases of supraventricular tachycardias with a maximum frequency of 240 beats per minute, which resolved until the end of hemodialysis. All other vital parameters were stable during the treatment.

**Figure 4:**
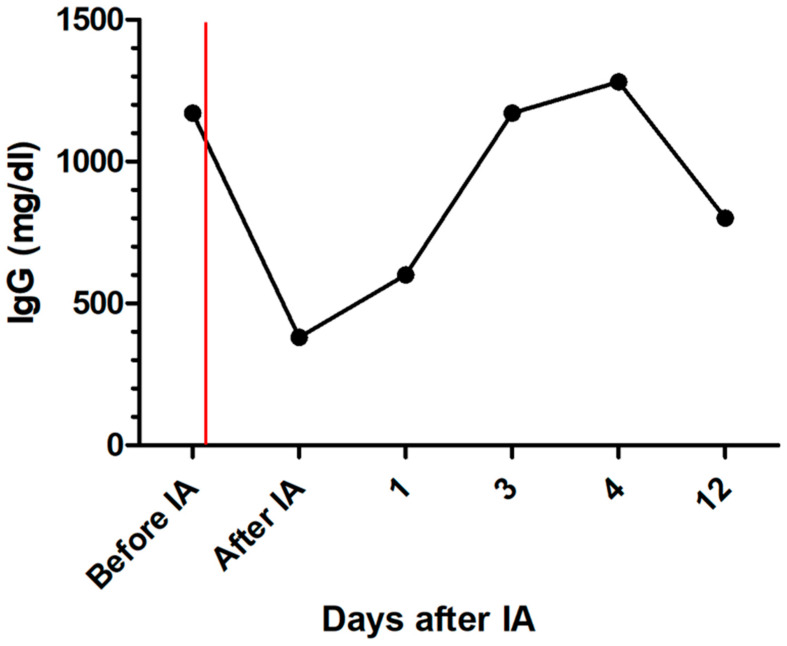
Development of total immunoglobulin G concentration during treatment of the dog with leishmaniosis and immunoadsorption; the red line marks the time point of immunoadsorption; IA = immunoadsorption; IgG = immunoglobulin G.

**Figure 5:**
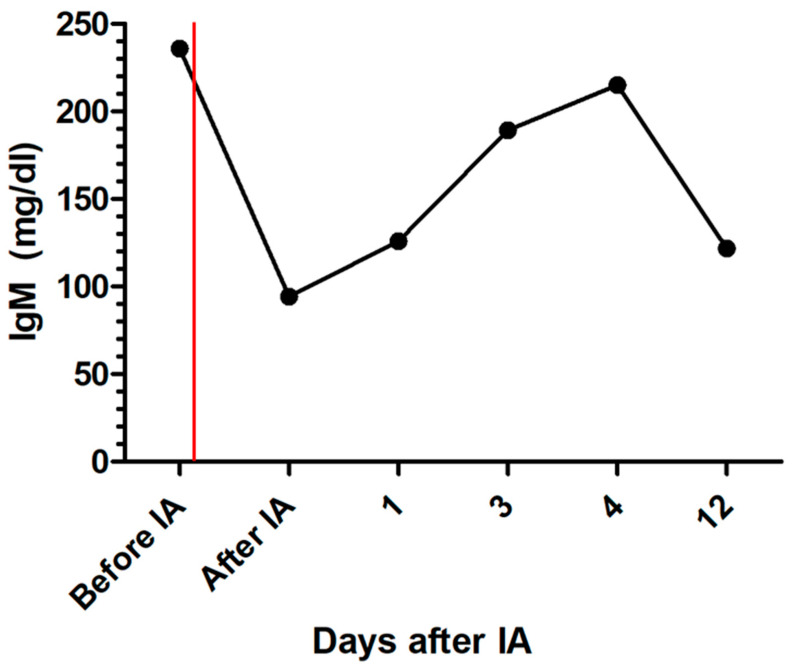
Development of total immunoglobulin M concentration during treatment of the dog with leishmaniosis and immunoadsorption; the red line marks the time point of immunoadsorption; IA = immunoadsorption; IgM = immunoglobulin M.

**Figure 6:**
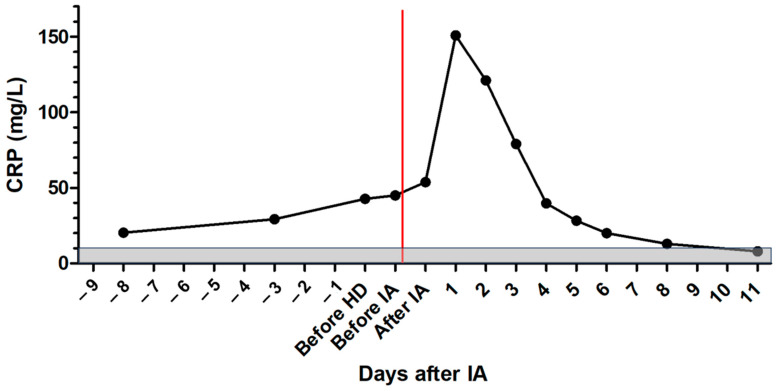
Course of CRP concentration in the patient with leishmaniosis during treatment; the gray area marks the reference range, the red line marks the time point of immunoadsorption; HD = hemodialysis; IA = immunoadsorption; CRP = C-reactive protein.

After IA, the dog was further treated with maropitant, esomeprazole, amoxicillin clavulanic acid, allopurinol, miltefosine, amlodipine, and clopidogrel. On the following days, creatinine concentration declined, and the patient improved significantly (Figure 1). Total IgG and IgM were measured and showed a reduction of 67.5% for IgG and 60.1% for IgM after IA. Immunoglobulins increased again on the following days (Figure 4 and Figure 5). C-reactive protein (CRP) was measured as a marker of inflammation. CRP concentration increased significantly after hemodialysis and IA and declined on the following days (Figure 6). The patient was discharged after two and a half weeks. After three and a half weeks, creatinine had stabilized to slightly above the reference range. UPC decreased to 1.74 on day 17 after IA.

Two years after the initial presentation, the patient was presented for a routine check-up and was still in excellent condition. Creatinine, UPC, and albumin were in the reference range.

## 3. Discussion

This is the first report of IA in a dog with leishmaniosis. The pathogenesis of canine visceral leishmaniosis mainly depends on the host’s immune response. Resistant dogs develop a Th1 immune response, resulting in the production of proinflammatory cytokines, which control the infection and associated inflammation by increasing the leishmanicidal activity of macrophages [4]. Susceptive dogs develop a systemic immune response dominated by Th2 cells, regulatory T cells, and regulatory B cells [4]. The cytokines produced by Th2 cells downregulate the protective Th1 immune response, promoting “inappropriate” humoral immune responses [6]. High concentrations of antibodies in combination with *Leishmania* antigen induce the formation of circulating immunocomplexes composed of aggregated *Leishmania* proteins, anti-*Leishmania* IgG and IgM, and, to a lesser extent, complement system fractions [4,7]. The deposition of the circulating immunocomplexes in different organs leads to inflammation and organ damage, clinically visible as uveitis, polyarthritis, vasculitis, and/or glomerulonephritis [4]. Human leishmaniosis patients show an increased excretion of lambda light chains in their urine [8]. Elevated concentrations of free light chains in the urine are known to cause renal damage in human medicine [9]. Elevated concentrations of free lambda light chains could also be detected in dogs with leishmaniosis, but the association with renal damage has not been demonstrated yet [10]. Glomerulonephritis and consecutive renal failure are the most severe complications in dogs with leishmaniosis and the most common reason for death in these patients [1,4].

Therefore, a major therapeutic goal should be the reduction of circulating antibodies in these patients. The potency of IA for the reduction of circulating immunocomplexes is well known in human medicine [5]. IA is successfully used for hematological, neurological, and other immunological systemic disorders. It was also shown to be effective in patients with immunological diseases affecting the kidney, such as systemic lupus erythematosus, and for the prevention of unwanted immunological reactions after renal transplantation [11]. In veterinary medicine, studies evaluating the potency of IA in different immunological diseases are lacking. There are only two case reports about IA in veterinary medicine. One case report showed a dog with immune-mediated hemolytic anemia successfully treated with IA, and the other one showed a dog with fulminant acquired myasthenia gravis successfully treated with IA [12,13]. The adsorption of IgG and IgM in the Ligasorb^®^ IA adsorber is unspecific. Therefore, the adsorption of *Leishmania* IgG and IgM antibodies in these patients is likely possible with IA. Therefore, IA can be a potential therapeutic approach to reduce circulating immunocomplexes and potentially fatal complications following the deposition of these immunocomplexes.

A previous case report of a dog with leishmaniosis treated with intermittent hemodialysis showed the potential benefit of extracorporeal blood purification techniques in such patients [14]. This dog was presented with proteinuria and acute kidney injury (AKI) grade V, as described by the guidelines of the International Renal Interest Society (IRIS) associated with canine leishmaniosis in the clinic. After being treated with three sessions of intermittent hemodialysis, the initial creatinine concentration of 683 µmol/L decreased to 211 µmol/L within 20 days. Creatinine concentration normalized over the following weeks after hospital discharge [14]. In the present case report, creatinine concentration after hemodialysis and IA decreased even faster than in the previous case report, from 666 µmol/L to 153 µmol/L within five days, with only one session of hemodialysis necessary for improvement. As the dog from the present study was already hospitalized for nine days and was treated with intravenous fluid therapy before hemodialysis and IA, azotemia could not be prerenal, and the decline in creatinine concentration was the result of a real improvement in renal function. The use of IA and the potential reduction of immunocomplexes are likely the reasons for the reduced need for hemodialysis sessions.

Another blood purification technique that is used in different immunological diseases is therapeutic plasma exchange (TPE). TPE was used to treat leishmaniosis, immune-mediated hemolytic anemia, or hyperviscosity syndrome in dogs [15,16]. IA might be a preferable technique to TPE as it does not require the replacement of plasma proteins [17]. For TPE, large volumes of canine plasma are necessary. For many clinics, canine plasma is not easy to produce or purchase and is therefore a limited resource. As IA does not need canine donor plasma, it could be a more easily available alternative to TPE.

Another blood purification technique used in immunological diseases is double filtration plasmapheresis (DFPP). DFPP is described as being useful for hyperviscosity syndrome and was effective in a case report of a dog with leishmaniosis [18,19]. In comparison to TPE, DFPP is a technique that selectively removes high-molecular-weight substances, including Ig and immunocomplexes, without the need for substitution fluids for the patient [19]. The case report suggests the use of DFPP in leishmaniosis patients, but its efficacy has so far not been proven by experimental studies. Therefore, it remains unknown so far which of the two techniques, IA and DFPP, is superior.

In the present case report, total Ig concentration was reduced by 67.5% for IgG and 60.1% for IgM. This reduction supports the theory of a potential benefit of IA in patients with immunocomplex glomerulonephritis due to leishmaniosis and corresponds with the improvement in clinical and laboratory parameters in this patient.

CRP was mildly elevated at presentation and increased significantly after hemodialysis and IA. However, on the following days, it decreased to the reference range. Processing the blood in an extracorporeal circuit and contact of the blood with the tubing system might activate the immune system and lead to the production of acute phase proteins [20]. This might be an explanation for the significant increase in CRP concentration after hemodialysis and IA.

UPC had increased after hospitalization and conservative medical treatment with intravenous fluid therapy, supportive medical treatment, and anti-*Leishmania* treatment. This indicates the deterioration of the disease and was a signal for insufficient therapy. After hemodialysis and IA, there was initially an increase in UPC, but it decreased 11 days after hemodialysis and IA. IA can only eliminate circulating immunocomplexes from the blood. Deposited immunocomplexes in the kidneys must be solved on their own. This might be a reason for delayed improvement in UPC, as UPC can only improve with the regeneration of kidney and glomerular function.

The survival time of the dog in the present case report was much longer than in other dogs with leishmaniosis and concurrent severe azotemia. Normally, severe azotemia is a bad prognostic factor and a major reason for euthanasia in dogs with leishmaniosis [21]. In a retrospective study about prognostic factors in dogs with leishmaniosis, the median survival time for dogs with IRIS stages 3 and 4 chronic kidney disease was around one month [22]. The dog in the present study showed excellent clinical condition and normal creatinine and UPC concentrations two years after IA.

A limitation of this case report is that the influence of IA on the course of disease cannot be objectively determined. It is possible that the patient’s kidney values would have been improved with hemodialysis alone. However, proteinuria, which was severe in this patient, is caused by the deposition of immunocomplexes and consecutive damage to the glomeruli by inflammation. It is unlikely that hemodialysis alone cures glomerulonephritis and proteinuria.

## 4. Conclusions

This case report shows the enormous potential of IA in patients with leishmaniosis. IA might be an opportunity to reduce antibodies and circulating immunocomplexes in dogs with leishmaniosis. Further studies are needed to support this hypothesis.

## Data Availability

The data presented in this study are available on request from the corresponding author.
